# Learning-based keypoint registration for fetoscopic mosaicking

**DOI:** 10.1007/s11548-023-03025-7

**Published:** 2023-12-09

**Authors:** Alessandro Casella, Sophia Bano, Francisco Vasconcelos, Anna L. David, Dario Paladini, Jan Deprest, Elena De Momi, Leonardo S. Mattos, Sara Moccia, Danail Stoyanov

**Affiliations:** 1 Department of Advanced Robotics, Istituto Italiano di Tecnologia, Genoa, Italy; 2 Department of Electronic, Information and Bioengineering, Politecnico di Milano, Milan, Italy; 3 Wellcome/EPSRC Centre for Interventional and Surgical Sciences (WEISS) and Department of Computer Science, University College London, London, UK; 4 The BioRobotics Institute and Department of Excellence in Robotics and AI, Scuola Superiore Sant’Anna, Pisa, Italy; 5 Fetal Medicine Unit, Elizabeth Garrett Anderson Wing, University College London Hospital, London, UK; 6 EGA Institute for Women’s Health, Faculty of Population Health Sciences, University College London, London, UK; 7 Department of Development and Regeneration, University Hospital Leuven, Leuven, Belgium; 8 Department of Fetal and Perinatal Medicine, Istituto Giannina Gaslini, Genoa, Italy

**Keywords:** Fetal surgery, Mosaicking, Twin-to-twin transfusion syndrome, Fetoscopy, Deep learning, Self-supervised

## Abstract

**Purpose:**

In twin-to-twin transfusion syndrome (TTTS), abnormal vascular anastomoses in the monochorionic placenta can produce uneven blood flow between the two fetuses. In the current practice, TTTS is treated surgically by closing abnormal anastomoses using laser ablation. This surgery is minimally invasive and relies on fetoscopy. Limited field of view makes anastomosis identification a challenging task for the surgeon.

**Methods:**

To tackle this challenge, we propose a learning-based framework for in vivo fetoscopy frame registration for field-of-view expansion. The novelties of this framework rely on a learning-based keypoint proposal network and an encoding strategy to filter (i) irrelevant keypoints based on fetoscopic semantic image segmentation and (ii) inconsistent homographies.

**Results:**

We validate our framework on a dataset of six intraoperative sequences from six TTTS surgeries from six different women against the most recent state-of-the-art algorithm, which relies on the segmentation of placenta vessels.

**Conclusion:**

The proposed framework achieves higher performance compared to the state of the art, paving the way for robust mosaicking to provide surgeons with context awareness during TTTS surgery.

**Supplementary Information:**

The online version contains supplementary material available at 10.1007/s11548-023-03025-7.

## Introduction

Twin-to-twin transfusion syndrome (TTTS) is a rare complication affecting 10–15% of monochorionic diamniotic pregnancies. TTTS is characterized by the development of unbalanced blood transfer from one twin (the donor) to the other (the recipient), through placental communicating vessels called anastomoses [1]. This shared circulation causes profound fetal hemodynamic unbalance and consequently severe growth restriction, cardiovascular dysfunction, hypoxic brain damage and death of one or both twins [2].

**Fig. 1:**
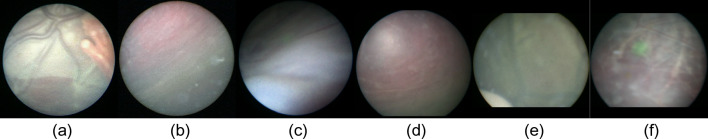
Main challenges of TTTS frames: **a** occlusions, **b**, **d**, **f** lack of anatomical structures (e.g., vessels), **c** poor frame texture, **e** non-planar view in case of anterior placenta

The recognized elective treatment for TTTS is selective laser photocoagulation of anastomoses originating in the donor’s placental territory. This treatment requires precise identification and laser ablation of placental vascular anastomoses [3]. Despite recent advancements in instrumentation and imaging for TTTS [4], residual anastomoses still represent a major complication [5]. This may be explained considering the challenges, from the surgeon’s side, of limited field of view (FoV) and constrained maneuverability of the fetoscope, especially for anterior placenta.

In this complex scenario, computer-assisted intervention (CAI) and surgical data science (SDS) methodologies [6] may be exploited to provide surgeons with mosaicking for FoV expansion. In the last years, mosaicking in fetoscopy has been widely investigated and several methods to tackle this task have been exploited, as in depth discussed in “Related work” section. Most of the work in the literature focuses on handcrafted features or relies on the detection of stable regions or anatomical landmarks, like blood vessels. However, none of the developed methodologies have been translated into surgical practice, and several challenges are still open. These challenges include poor visibility due to amniotic fluid turbidity, low resolution of fetoscopic images, occlusions by surgical tools and fetuses (Fig. 1a), lack of anatomical structures (Fig. 1b, d, f) to be used as reference for frame registration, poor frame texture (Fig. 1c) and distortion introduced by non-planar views due to fetoscope camera orientation, especially in case of anterior placenta (Fig. 1c, e). [7]

With this work, we aim to contribute to the advancement of the state of the art in FoV expansion for TTTS by investigating, with a comprehensive study with six videos (1450 frames) acquired during actual surgery [8], and the research hypotheses are as follows:

- Hypothesis 1 (H1): Keypoint learning can tackle the challenges typical of fetoscopic videos acquired during TTTS surgery and provide robust keypoints for mosaicking without relying on the segmentation of anatomical structures in the FoV.
- Hypothesis 2 (H2): Mosaicking performance can be boosted by filtering irrelevant keypoints using semantic information and rejecting inconsistent homographies.

### Contribution

In this paper, we propose a learning-based framework for the robust detection of keypoints with the aim to register consecutive frames acquired during TTTS surgery and accomplish mosaicking for FoV expansion in fetoscopy. Our framework does not depend on anatomical landmark segmentation for frame registration. However, when either fetuses or surgical tools are present within the FoV, their segmentation is used for irrelevant keypoints rejection. The contributions of this work can be summarized as follows:

1. Development of a new framework for learning-based FoV expansion in TTTS fetoscopy videos, which features a self-supervised training strategy for detecting robust keypoints.
2. Development of a filtering strategy for (i) removing irrelevant keypoints by exploiting keypoints semantic from surgical scene segmentation, and for (ii) filtering out inconsistent homographies.

**Fig. 2:**
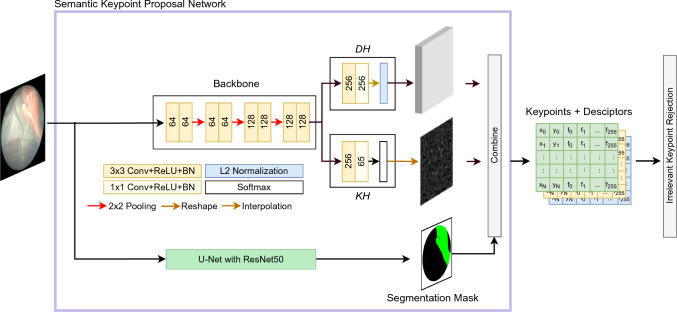
Overview of the semantic keypoint proposal network. *KH* and *DH* are the keypoint and keypoint descriptor head, respectively. Irrelevant keypoint rejection relies on semantic segmentation performed by the U-Net with ResNet50 backbone from [8]. The overall output is a set of keypoints, their descriptors and class

In this paper, we introduce a method that uses learned keypoints and use segmentation to assign them semantic labels that allow us to discriminate between useful and irrelevant keypoints for image registration. To the best of our knowledge, this work is the first to investigate the potential of learned semantic keypoints to achieve mosaicking in fetoscopy. We also conduct an extensive comparison with the state of the art and present an ablation study to identify the optimal configuration of our framework. The code is made available^1^ for reproducibility.

## Related work

Handcrafted local feature descriptors, such as SIFT and ORB, have been commonly used for image matching, also in medical field, due to the associated low computational cost. For example, the work in [9] uses SIFT descriptor to extract local features, optimizing feature selection based on a Voronoi diagram for retinal image mosaicking. SURF descriptors are used for real-time bladder mosaicking in fluorescence endoscopy in [10], while in [11], the authors use a SIFT-based zone matching method specifically designed for endoscopic images. Similarly, the work in [12] proposes an improved feature point algorithm for endoscopic image matching based on the SIFT descriptor. First attempts to use local descriptors for mosaicking in fetoscopy include [13, 14]. These methods have only been validated on synthetic phantoms or ex vivo placental sequences. However, when it comes to in vivo images, researchers showed that traditional local feature descriptors are not able to tackle the complexity of intraoperative images [7, 15].

The work in [16] followed a different approach by minimizing the photometric consistency between frames, showing promising results with in vivo fetoscopy data. However, the computation time to process a frame pair is a major bottleneck and may not be compatible with real-time mosaicking.

More recently, deep learning-based algorithms have been proposed to try to improve the performance of fetoscopy mosaicking while keeping the computational cost low. In [17], stable regions identified by a convolutional neural network (CNN) are used as a prior for frame registration. The approach is tested on phantoms only. The work in [18] uses HomographyNet to perform pair-wise homography estimation. The validation is performed on a single in vivo sequence. In [8], the authors show that placental vessels provide unique landmarks to compute homography. While obtaining accurate vessel segmentation might be considered an affordable challenge [7], this approach fails whenever vessels are not clearly visible. The work in [19] proposes to use pixels flow field for homography estimation using FlowNet, thus enabling mosaicking without relying on vessels. However, FlowNet requires constant brightness and robust texture [20], which cannot always be guaranteed in fetoscopic frames.

In the field of natural-image analysis, current researches focus on learned keypoints and local descriptors, showing promising results. The work in [21] proposes a siamese network which relies on L2 distance between patches to select those images that are challenging to match during training in order to learn better descriptors. Similarly, the authors in [22] propose a network named L2-Net that outperform traditional descriptors. GeoDesc proposed in  [23] enforces geometrical consistency during training to learn stable descriptors in images from multiple views.

**Fig. 3:**
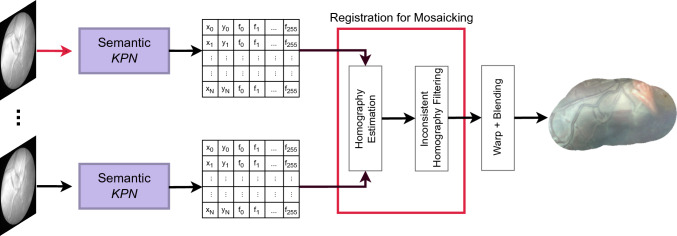
Overview of the proposed mosaicking framework. The keypoint proposal network (*KPN*) computes keypoints that are then filtered, according to their semantics, to reject irrelevant keypoints. Registration for mosaicking is performed to register consecutive fetoscopy frames. Warping and blending are performed for visual purposes

Inspired by these researches, we decided to exploit learned keypoints and local descriptors. This opens up new possibilities in translating these techniques to fetal surgery as keypoints and descriptors are widely used in image-based navigation systems [24]. However, learning keypoints requires supervision [25, 26], which is not trivial to be generated in fetoscopy due to the complexity in the definition of stable keypoints and, thus establishing the ground truth. To address this issue, in our framework, we rely on self-supervised learning as a solution [27] (Fig. 2).

## Proposed method

Our proposed framework consists of two main modules, (i) a semantic keypoint proposal network (*KPN*) (“SuperPoint: the keypoint proposal network” section) for keypoints learning, (ii) an irrelevant keypoint rejection, and (iii) registration for mosaicking (“Registration for mosaicking” section), for estimating transformation, as homography, from the keypoints and filtering inconsistent homographies. The overall framework is shown in Fig. 3.

### SuperPoint: the keypoint proposal network

The semantic keypoint proposal network (*KPN*) is a CNN based on SuperPoint [25] (Fig. 2) and consists of keypoint proposal computation (“Keypoint proposal computation” section), trained following the strategy described in “KPN training” section, and irrelevant keypoint rejection using semantic information from segmentation (“Semantic keypoint rejection” section).

#### Keypoint proposal computation

*KPN* consists of a VGG-16 backbone for feature extraction, followed by two heads, the keypoint head (*KH*) for the detection of candidate keypoints, and the descriptor head (*DH*) for computing keypoint descriptors. *KH* outputs a dense point map, with the same size as the input frame, where the value of each pixel refers to the probability of that pixel of being a keypoint. *DH* outputs a L2-normalized descriptor vector for each candidate keypoint.

#### KPN training

We train the *KPN* in four steps. To account for the lack of annotated TTTS frames, we initially trained *KPN* without *DH* on the synthetic shapes dataset presented in DeTone et al. [25]. Each pair consists of images with size $448 \times 448$ pixels containing simple polygons and associated keypoints. A robust keypoint should be covariant with respect to visual conditions and camera motion transformation, thus to encode this property during *KPN* training, we increase the dataset by applying (i) perspective distortions (i.e., homographic augmentation) to model different camera views and (ii) brightness and contrast (i.e., photometric augmentation).

As second step, we fine-tune *KPN* on natural images from MS-COCO 2014 dataset [28]. In this case, we follow a self-supervised training strategy to account for the dataset lack of keypoint annotation. We infer the KPN trained at the previous step to generate the pseudo-ground truth. We apply homographic and photometric augmentation during training to increase the variability of the dataset and implicitly filter inconsistent keypoints.

In the third step, we generate the pseudo-ground truth on a subset of our TTTS dataset with a leave-one out schema (i.e., the patient used for testing is excluded from the training set), using *KPN* from the second step and then we perform the fine-tuning.

Performing several iterations of this procedure further improve the *KPN* performance. In the last step, we still use the TTTS subset from the third step to compute the pseudo-ground truth. This pseudo-ground truth is used to train the whole *KPN*. We limit the parameter range for homographic augmentation to be consistent with the fetoscope camera model.

**Table 1:** Characteristics of the extended fetoscopy placenta dataset presented in [8]

Video ID	Frame number	Frame resolution	Placenta position
		[Pixels]	
1	400	470 $\times$ 470	Posterior
2	300	540 $\times$ 540	Posterior
3	150	550 $\times$ 550	Anterior
4	200	640 $\times$ 640	Posterior
5	200	720 $\times$ 720	Anterior
6	200	720 $\times$ 720	Posterior

For all the steps, we use the loss function $\mathcal {L}_{ KPI }$ defined as:

1 $$\begin{aligned} \mathcal {L}_{ KPI }=\mathcal {L}_{KP}+\mathcal {L}_{KP}'+\lambda \mathcal {L}_{D}(D,D') \end{aligned}$$

where $\mathcal {L}_{KP}$ is the cross-entropy loss computed over the keypoint map generated by *KH* and its groundtruth, and $\mathcal {L}_{KP}'$ is the loss computed on the warped keypoint map generated by *KH* after image warping with a random homography. In the fourth step, DH is trained along with *KPN*, thus we added the hinge loss between descriptors from the original image and those from the warped image ($\mathcal {L}_{D}(D,D')$) to the overall loss, weighted by the term $\lambda$. $\lambda$ is initially set to 0.0001 and then adjusted during training to balance the effect $\mathcal {L}_{D}(D,D')$ term that, especially in the first training epochs, has largely negative values.

#### Semantic keypoint rejection

We noticed experimentally that *KPN* finds keypoints on structures, such as fetuses and surgical tools. These structures can move in the foreground, and their registration could break the nearly planar assumption that we are considering in fetoscopic placenta images registration leading to inconsistent camera movement estimation. To reject irrelevant keypoints, we filter out keypoint proposals according to semantic segmentation masks removing all those keypoints detected on fetus and surgical tool. The semantic segmentation is obtained using the U-Net with ResNet50 backbone model trained on the annotated data for segmentation from the “FetReg2021 Challenge Dataset” [7].

### Registration for mosaicking

In this section, the registration pipeline for mosaicking will be described. Once the image-pair keypoints are computed, the latest block of our framework will perform homography estimation (“Homography estimation” section) and filtering (“Inconsistent homography filtering” section).

#### Homography estimation

Assuming *KPN* to be robust, we design a simple frame-pair registration pipeline to achieve fast registration at low computational cost. The *KPN* identifies all potential keypoints within a frame. If multiple keypoints are detected in a small neighborhood ($4 \times 4$ window), only those with the highest probability are kept, thanks to non-maximum suppression (NMS). The subsequent semantic segmentation step filters out irrelevant keypoints. The keypoints that remain are ranked by probability, and only the top 1000 are used for matching and homography computation. We approximate registration as affine transformations, following the considerations in Bano et al. [8]. The homography of two consecutive frames is estimated using RANSAC and least squares optimization.

#### Inconsistent homography filtering

We can assume that homographies should not reflect large displacement, rotation or scaling (i.e., displacement $\pm 8$ pixels, rotation $\pm 15$ degrees and scaling $\pm 5\%$), as we register consecutive frames. We take inspiration from Bano et al. [18] to filter out any homography that does not reflect this assumption. We perform singular value decomposition on each estimated homography to extract rotation, scale and translation parameters. When one of these parameters exceeds a threshold defined experimentally, the second frame in the pair to be registered is discarded, and the registration with the next frame is attempted. This procedure is reiterated for five frames and, in case of failure, mosaicking computation ends.

## Experimental setup

### Dataset

We trained and validated our framework using a leave-one out schema at patient level on the extended version of the “Fetoscopy Placenta Dataset” published in [8] for fair comparison with the literature. The overall dataset consists of 1450 frame from six different in vivo TTTS fetoscopy procedures. Main characteristics of the dataset are summarized in Table 4.

Videos differ in terms of resolution, intraoperative environment, artifacts and lighting conditions. Two videos present the anterior placenta. While in posterior placenta, the scene can be considered nearly planar, the use of 30-degrees fetoscope for anterior placenta introduces non-planar view and more challenges to mosaicking [29, 30].

**Table 2:** Ablation study—summary

	*KPN*	Irrelevant keypoint	Inconsistent homography
		rejection	filtering
E0	X^∗^		
E1	X		
E2	X	X	
Proposed	X	X	X

*KPN* keypoint proposal network (“Keypoint proposal computation” section). ^∗^For E0, we test SuperPoint trained on MS-COCO 2014 dataset without any fine-tuning on fetoscopy data

**Table 3:** Quantitative results for the six tested in vivo fetoscopy videos

	Video 1	Video 2	Video 3
SIFT	$0.662 \pm 0.115$	$0.732 \pm 0.120$	$0.749 \pm 0.279$
Bano et al. [8]	$\mathbf {0.757 \pm 0.081}$	$\mathbf {0.788 \pm 0.050}$	$0.839 \pm 0.208$
Pre-trained SuperPoint (E0)	$0.528 \pm 0.247$	$0.202 \pm 0.264$	$0.219 \pm 0.266$
Vanilla SuperPoint (E1)	$0.731 \pm 0.116$	$0.740\pm 0.079$	$0.809 \pm 0.174$
Semantic *KPN* (E2)	$0.730 \pm 0.112$	$0.743\pm 0.071$	$0.813 \pm 0.172$
Proposed	$0.750 \pm 0.081$	$0.766 \pm 0.048$	$\mathbf {0.884 \pm 0.075}$
	Video 4	Video 5	Video 6
SIFT	$0.660 \pm 0.347$	$0.5164 \pm 0.402$	$0.485 \pm 0.389$
Bano et al. [8]	$0.745 \pm 0.257$	$0.890 \pm 0.070$	$0.861 \pm 0.205$
Pre-trained SuperPoint (E0)	$0.322 \pm 0.362$	$0.341 \pm 0.284$	$0.209 \pm 0.336$
Vanilla SuperPoint (E1)	$0.801\pm 0.111$	$0.829\pm 0.091$	$0.817 \pm 0.076$
Semantic *KPN* (E2)	$0.818 \pm 0.111$	$0.832 \pm 0.090$	$0.817 \pm 0.073$
Proposed	$\mathbf {0.870 \pm 0.125}$	$\mathbf {0.897 \pm 0.012}$	$\mathbf {0.909 \pm 0.021}$

The *s* with *n* = 5 frames is reported in terms of mean ± standard deviation

Higher value of *s* for each video are highlighted in bold

### Implementation details

Our framework is implemented in TensorFlow 1.15 and trained on two NVIDIA A100 40GB, using ADAM optimizer and a learning rate of $10^{-3}$. For training the semantic *KPN* following the strategy described in “KPN training” section, in the first three training steps, we set a batch size of 64, while a batch size of 8 is used for the last step. For the 4 steps, we set a number of iteration equal to 180,000, 60,000, 20,000 and 12,000, respectively (Table 1).

### Performance metrics

We measure the performance of our framework using the structural similarity index measure (SSIM) over a number (*n*) of frames, with $n \in [1, 5]$, for fair comparison with Bano et al. [8]. We call this metric *s*.

**Fig. 4:**
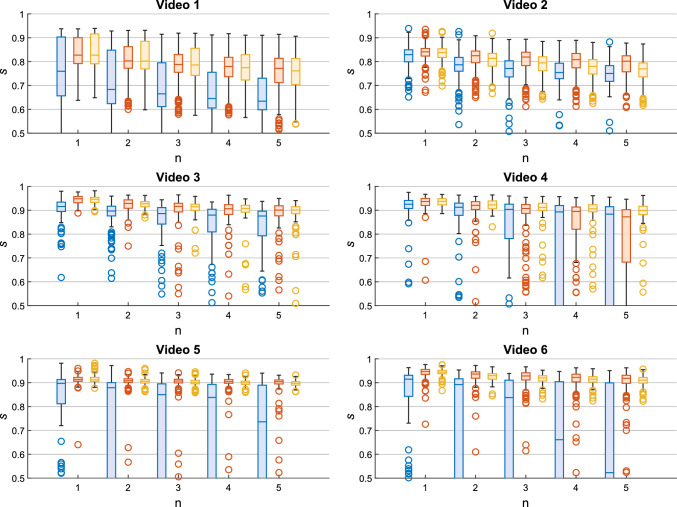
Boxplots of *s* over *n* frames (with *n* in range [1–5]) obtained with (blue) SIFT, (red) [8] and (orange) the proposed framework

Given a source ($I_{i}$) and a target ($I_{i+n}$) frame, and a homography transformation (${H}_{i \rightarrow i+n}$) between $I_{i}$ and $I_{i+n}$, for every *i*-th frame in the TTTS sequence *s* is defined as:

2 $$\begin{aligned} s_{i \rightarrow i+n} =\textrm{sim}\big (w\big (\tilde{I}_{i},H_{i \rightarrow i+n}\big ),\tilde{I}_{i+n}\big ) \end{aligned}$$

where $\textrm{sim}()$ is the standard formula for SSIM, *w* is the warping operator, and $\tilde{I}$ and $\tilde{I}_{i+n}$ are smoothed versions of *I* and $I_{i+n}$, respectively. $\tilde{I}$ and $\tilde{I}_{i+n}$ are obtained by applying $9\times 9$ Gaussian filtering with standard deviation of 1.5. This makes *s* robust even in the presence of amniotic fluid particles and fetoscopy-image noise. When exploring the vascular network, the fetoscope mainly makes small movements with consecutive frames almost completely overlapped. In this scenario, similarity metrics on low texture images are not useful for identifying errors between registration methods. We computed *s* with *n* larger than 1 to consider a wider frame interval and thus, make *s* more sensitive to registration errors. In case a frame is discarded, the metric will be computed considering an identity transformation. As additional performance metric, we also provide the root-mean-square error (RMSE) computed between each frame and its geometrically augmented version, using the same settings from homographic adaptation, to compute a synthetic groundtruth. We used the formulation of 4-point homography as described in [8], hence the RMSE is given by:

3 $$\begin{aligned} e _{R} = \sqrt{\frac{1}{4}\Sigma _{i=1}^{4}{\Big ([(\Delta u_{i} - \Delta \hat{u}_{i})^2 + (\Delta v_{i} - \Delta \hat{v}_{i})^2\Big )}} \end{aligned}$$

where $\Delta u$ and $\Delta v$ are the groundtruth displacements of the four corners, and $\Delta \hat{u}$ and $\Delta \hat{v}$ are the estimated displacements.

For qualitative evaluation, the registered frames are blended together using the Mertens–Kautz–Van Reeth exposure fusion algorithm [31] to tackle the non-uniform light exposure of the FoV along the fetoscopic video sequence.

### Comparison with the literature and ablation study

We compared our framework with SIFT, which is a standard feature extractor used for mosaicking [13, 14]. We further compared our framework with Bano et al. [8], which relies on deep learning for mosaicking and is the best performing methods in the state of the art. For all our competitors, we replace any discarded homography with an identity matrix to preserve the frame numerical consistency across the methods. The ablation study characteristics are summarized in Table 2.

As ablation study, we considered the following experiments:

- Experiment 0 (E0): SuperPoint pre-trained on MS-COCO 2014 dataset, without any fine-tuning on fetoscopy data.
- Experiment 1 (E1): Vanilla *KPN*, as described in “Keypoint proposal computation” section. Here, both irrelevant keypoint rejection and inconsistent homography filtering are excluded.
- Experiment 2 (E2): Semantic *KPN*, as described in “SuperPoint: the keypoint proposal network” section. Only inconsistent homography filtering is hence excluded.

For E2, we further investigate the performance obtained on an extended version of the dataset presented in “Dataset” section. This extended version consists of the same six videos, but each video has an extended length (avg sequence length = 546 ± 237 frames). This allows us to evaluate the benefits of introducing homography filtering for longer video sequences.

**Fig. 5:**
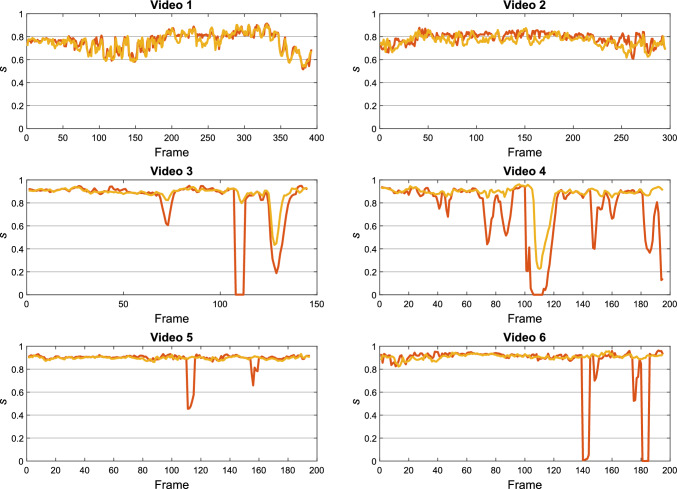
Plot of *s* with *n* = 5 computed for all video length. The curves refer to (red) [8] and (orange) the proposed framework

## Results

The average *s* values with *n* equal to 5 obtained with SIFT, the work in [8] and the proposed framework are reported in Table 3. SIFT shows the lowest performance, as it fails in retrieving keypoints for mosaicking for several frames in all the six videos. This is in agreement with similar findings in the SDS/CAI field reported by Liu et al. [32].

**Fig. 6:**
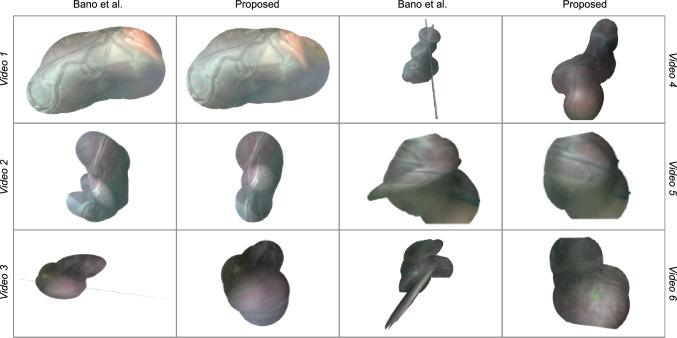
Mosaics obtained from the six TTTS videos using the method from Bano et al. [8] and the proposed framework. Results refer to the dataset presented in “Dataset” section

For Video 1 and Video 2, where vessels are clearly visible and lens distortion is small, we obtained *s* with *n*=5 equal to $0.750 \pm 0.050$ and $0.766 \pm 0.048$, respectively. These results are comparable to that of Bano et al. [8] ($0.757 \pm 0.081$ and $0.788 \pm 0.050$, respectively). Hence, the work in Bano et al. [8] slightly outperforms the proposed framework for Video 1 and Video 2 by only 0.007 and 0.022, respectively. This was not true when considering the other videos, where the average *s* was the highest for the proposed framework, which also granted the lowest standard deviation. The proposed framework overcomes Bano et al. [8] by at least 0.007 (video 5), with the highest difference for video 6 (0.045) and video 4 (0.125).

These findings are also confirmed by $$ e _{R}$$, where Bano et al. [8] and the proposed method have comparable median values on video 1 (0.108 and 0.112, respectively, video 2 (0.123 and 0.120, respectively) and video 3 (0.101 and 0.094, respectively). The proposed method achieved lower $$ e _{R}$$ in videos 4 to 6 ($$ e _{R}$$ (0.099, 0.092 and 0.097) compared to Bano et al. [8] ($$ e _{R}$$ (0.145, 0.122 and 0.171).

Figure 4 reports the value of *s* at different *n* obtained with SIFT, the work in Bano et al. [8] and the proposed framework for the six tested videos. The proposed framework consistently outperformed the competitors for every *n* for videos from 3 to 6. In the first two videos, the performance of the proposed framework were comparable to that of Bano et al. [8].

**Table 4:** Quantitative results for the extended dataset with longer fetoscopy sequences

	Video 1	Video 2	Video 3
E2	$0.735 \pm 0.154$	$0.710 \pm 0.014$	$0.811 \pm 0.210$
Proposed	$0.751 \pm 0.098$	$0.771 \pm 0.072$	$0.886 \pm 0.091$
	Video 4	Video 5	Video 6
E2	$0.810 \pm 0.140$	$0.802 \pm 0.320$	$0.791 \pm 0.164$
Proposed	$0.872 \pm 0.132$	$0.896 \pm 0.022$	$0.901 \pm 0.051$

Figure 5 shows the trend of *s* with *n* = 5 in time for the proposed method and Bano et al. [8]. The trend of *s* for Bano et al. [8] in videos from 3 to 6 shows drops in correspondence of wrong homography estimation. This happens to a lesser extent also for the proposed framework, but only in videos 3 and 4.

The quantitative analysis presented in Fig. 5 may also be appreciated from the qualitative examples shown in Fig. 6, where the proposed framework achieves good-quality mosaicking for all the tested videos also when vessels are not visible.

The pre-trained SuperPoint (E0) achieves *s* over $n=5$ frame of 0.331, showing lower performance than also SIFT. E1, which aims at assessing the performance of vanilla *KPN* alone, hence excluding both inconsistent keypoint rejection and homography filtering. In this experiment, we achieve an average *s* of 0.788, with a lost of 0.058 over the proposed framework.

With our ablation study E2, which aims at evaluating the benefit of introducing inconsistent keypoint rejection after the *KPN*, we achieve an average *s* with $n=5$ of 0.848. Despite the relatively small difference (0.064) in the performance achieved by our framework over E2, inconsistent homography filtering allows us to lower the drift in the mosaic and mitigate tracking loss in challenging videos, where images are strongly underexposed or whether noisy keypoints are computed (e.g., due to particles). However, when processing the extended version of this dataset with longer sequences, our results improve by $3\%$ when adding homography filtering, as shown in Table 4.

**Fig. 7:**
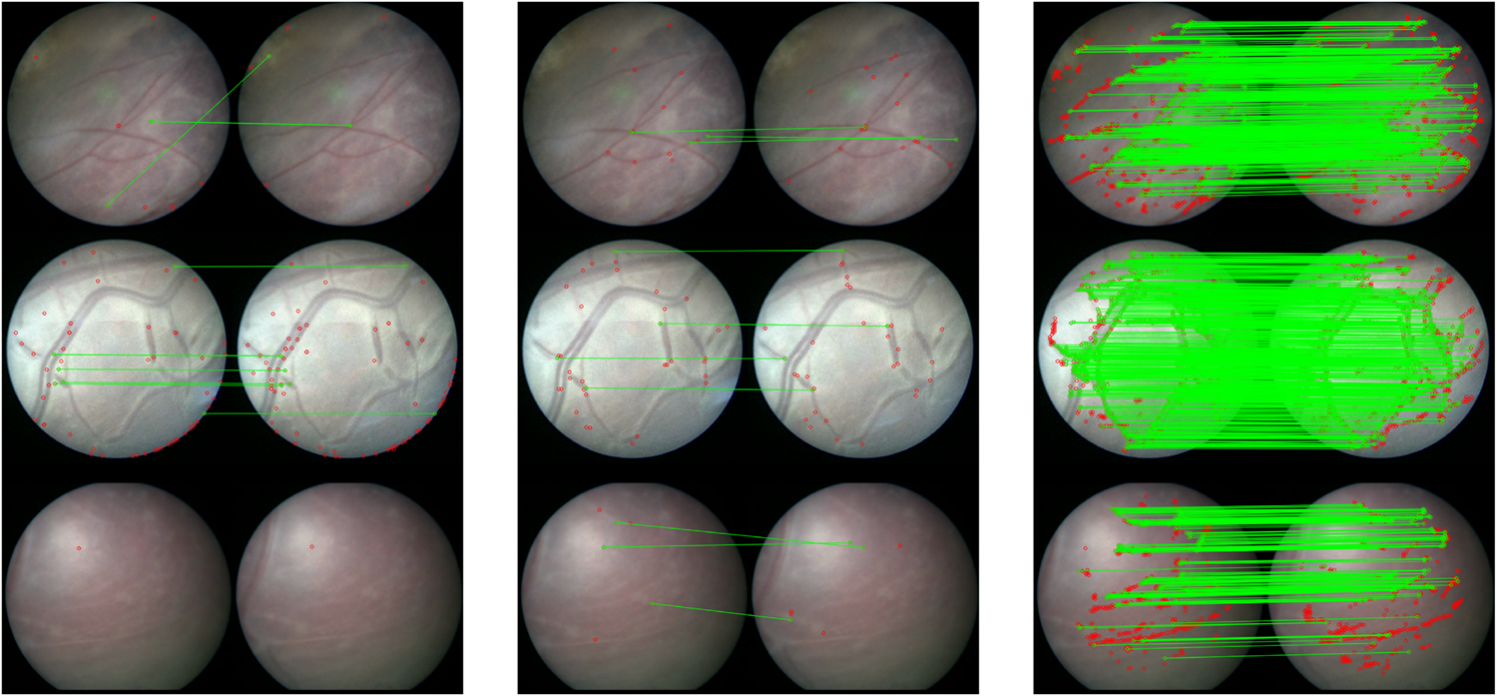
Visual comparison of keypoint computation from (left) SIFT, (middle) E0 and (right) proposed framework

## Discussion and conclusion

In this work, we proposed a mosaicking framework to perform FoV expansion in fetoscopy videos using learning-based keypoints. Going beyond the current state of the art, our framework does not rely on any prior vessel segmentation and instead uses a self-supervised keypoints detector which makes it robust when registering frames where vessels are not clearly visible or their segmentation is not accurate. We instead use semantic information from segmentation to filter out irrelevant keypoints and propose a simple yet effective strategy to discard inconsistent homographies.

To test our first research hypothesis (H1), we applied our proposed framework on six clinical videos from TTTS surgeries. We also compared the proposed framework with state-of-the-art approaches for fetoscopic mosaicking (Table 4), showing that our method performs well when others fail. From our experiments (Fig. 7), SIFT was not always able to find a sufficient number of keypoints to compute homography. This can be explained considering that SIFT is not robust in case of images with low contrast and texture, as in vivo fetoscopic images.

For comparison with Bano et al. [8], the absence of placenta vessels in a number of consecutive frames (Video 3, 4 and 5) hampered the registration process, while this not happened with our framework. Moreover, from Video 3 to Video 6, the placenta surface is not perfectly planar, and the lens distortions is more evident and camera moves along different planes to scan it entirely. Nonetheless, the proposed framework did not fail in providing good-quality mosaicking.

Our second research hypothesis (H2) was focused on assessing the benefits of including irrelevant keypoint rejection using semantics and inconsistent homography filtering. When analyzing *s* over the entire sequences (Fig. 5), our framework showed a lower number of drops in *s* than Bano et al. [8]. However, small drops were present in Video 3 and Video 4. This may be due to underexposed frames where keypoint estimation is particularly challenging. However, as the amount of underexposed frames was reasonably small, the inconsistent homography filter was able to tackle the challenge.

The benefit of adding semantic information for inconsistent homography filtering was specifically useful in long-range videos, as shown in the supplementary materials. We explain this improvement considering that the extended dataset includes further challenges (i.e., field-of-view occlusions, faster fetoscope movements and extreme change in illumination).

Quantitative and visual evaluations suggest that the proposed framework may provide computer-assisted interventional support for TTTS procedures by providing a robust method to increase the FoV facilitating the localization of abnormal placental vascular anastomoses. We also identified several additional advantages of the proposed approach. *KPN* does not require annotations and thus performance can be improved with additional data in the future at very limited cost. The use of keypoints and descriptors enables the integration with localization and mapping frameworks (e.g., SLAM), paving the way for the design and implementation of a navigation system for fetal surgery. Furthermore, the low computational cost and the close to real-time performance of our framework would ease its clinical translation. This may have a positive impact, by reducing surgeons’ mental workload and, as a consequence, potentially reducing patients’ risks and lowering surgery duration.

Possible limitations of the proposed framework may be encountered during sudden changes in illumination or in images with extreme exposure. In these circumstances, it may not be possible to detect enough keypoints to compute homography. In such case, the inconsistent homography filter may mitigate the failure of mosaicking only if this happen within a few frames. Another possible limitation of this framework is the absence of maternal breath handling. Although this does not compromise the usability of the generated mosaic, it may introduce some minor distortions. Future investigations will explore the performance of this framework with more patients and would extend this framework to deformable registration and integration with refinement and localization strategies [33] to achieve a complete navigation framework for fetal surgery. Additionally, we identified that determining a reliable metric to evaluate when a mosaic is good is non-trivial. The problem of finding proper metrics for machine learning task in medicine has recently garnered significant attention within the surgical data science community [34, 35], and we plan to foster this topic in future investigations.

### Supplementary Information

Below is the link to the electronic supplementary material.

Supplementary file 1 (m4v 13831 KB)

## Footnotes

1 https://github.com/alessandrocasella/learningmosaicking.
